# Patients’ expectations versus doctors’ expectations in antiretroviral therapy

**DOI:** 10.1186/1471-2334-14-S7-O7

**Published:** 2014-10-15

**Authors:** Alexandra Maria Largu, Carmen Dorobăț, Liviu Prisăcariu, Cristina Nicolau, Carmen Manciuc

**Affiliations:** 1 Infectious Diseases Hospital “Sf Parascheva”, Iaşi, Romania

## Background

Antiretroviral therapy (ART) is essential in maintaining a low HIV viral load, which allows the immune system to function properly and assures a qualitative life style for HIV positive patients. However, low rates of adherence to ART are still registered, in spite of all efforts. This paper aims to uncover what patients really expect from ART, and also what infectious diseases doctors expect from a patient’s ART regimen, thus exploring an important side of adherence to ART.

## Methods

From January to July 2014 we have conducted a qualitative study regarding both patients’ and doctors’ expectations regarding ART. We interviewed 30 patients and 4 doctors. We used semi-structured interviews that were conducted in the Psychosocial Compartment of the HIV/AIDS Regional Center in Iaşi.

## Results

The patients we interviewed came from all 6 counties in the Moldova area. Age varied from 16 years to 59 years; 55% were female and 45% male. 30% came from a rural area.

The most common expectations that patients have regarding ART are: “to help me live”, “not to make me feel sick”, “to be easy to take (not to big, not a lot)”, “not to show on the outside what I have on the inside”.

The infectious diseases doctors that we interviewed work in the HIV/AIDS Regional Center in Iaşi. Their expectations regarding an ART regimen for patients were: “to reduce HIV viral load”, “to increase CD4 cell count” and “to have minimal impact on the proper functioning of other organs”.

## Conclusion

Patients’ expectations from ART show the importance they give to the impact that the therapy has on their overall quality of life, especially on their body’s reaction to medication. Multiple side effects – nausea, diarrhea, headaches, skin rash, etc., or a large number of pills to take are, for patients, signs that the therapy did not meet their expectations and therefore a reason to stop taking it.

Doctors expect pragmatic results from ART, focusing on the medical aspects. Their perspective is centered less on the overall quality of life of the patient, and that is why often the doctor-patient relationship has to suffer.

In order to assure adherence to the ART it is important to explore both the doctor and the patient’s perspective and to find ways to find a common ground in building a healthy relationship. A healthy mediation is provided by the psychologist working directly with both parties.

